# Twenty-year cytogenetic and molecular follow-up of a patient with ring chromosome 15: a case report

**DOI:** 10.1186/1752-1947-6-283

**Published:** 2012-09-07

**Authors:** Roberta S Guilherme, Vera de FA Meloni, Sylvia S Takeno, Renata Pellegrino, Decio Brunoni, Leslie D Kulikowski, Maria I Melaragno

**Affiliations:** 1 Department of Morphology and Genetics, Universidade Federal de São Paulo, Rua Botucatu 740, São Paulo 04023-900, Brazil; 2 Department of Psychobiology, Universidade Federal de São Paulo, Rua Botucatu 740, São Paulo 04023-900, Brazil; 3 Department of Pathology, School of Medicine, LIM 03, Universidade de São Paulo, Avenida Dr. Enéas Carvalho de Aguiar 255, São Paulo 05403-000, Brazil

## Abstract

**Introduction:**

Ring chromosome 15 is a rare disorder, with only a few over 40 cases reported in the literature. There are only two previous reports of cases where patients with ring chromosome 15 have been followed-up.

**Case presentation:**

We report here on the 20-year clinical and cytogenetic follow-up of a patient with a ring chromosome 15. Our patient, a Caucasoid Asian woman, presented with short stature, microcephaly, minor dysmorphic features, hyperextensible knees, generalized hirsutism, café-au-lait and small hypochromic spots spread over her face and the front of her chest and abdomen, dorsolumbar scoliosis and mild intellectual disability. She was followed-up from the age of eight to 28 years. When she was 27 years old, she was reported by her mother to present with compulsive overeating and an aggressive mood when challenged. Karyotyping revealed that the majority of her cells harbored one normal chromosome and one ring chromosome. Silver staining revealed the presence of the nucleolar organizer region in the ring chromosome. Ring loss and/or secondary aberrations exhibited a slight increase over time, from 4.67% in 1989 to 7.67% in 2009, with the presence of two monocentric rings, cells with interlocked rings, a dicentric ring, and broken or open rings. A genome-wide array technique detected a 5.5Mb deletion in 15q26.2.

**Conclusions:**

We observed that some phenotypic alterations in our patient can be associated with gene loss and haploinsufficiency. Other features may be related to different factors, including ring instability and epigenetic factors.

## Introduction

Ring chromosome 15 [r(15)] is a rare disorder that was first described by Jacobsen in 1966. Since then, more than 40 patients with r(15) have been reported in the literature. The common features found in these patients are: severe pre- and postnatal growth delay, low birth weight, triangular face, hypertelorism, microcephaly, intellectual disability, clinodactyly and brachydactyly of the fifth finger, small hands and feet, and multiple hyperpigmented or depigmented spots. Severe phenotypes can also be observed, presenting with congenital heart defects and renal anomalies. Some patients present with hypotonia, hydrocephaly, micrognathia, retinal abnormalities, ear anomalies, club feet, café-au-lait spots, behavioral disorders and speech delay. Male patients may present with cryptorchidism, azoospermia, hypogonadism or hypogenitalism and in most of the cases they are sterile. Female patients seem to present with normal sexual development and gonadal function, but uterine hypoplasia has been reported in some cases [1].

A precise genotype-phenotype correlation is difficult to establish in these patients, because of differences in breakpoints, level of mosaicism and ring instability that result in a variable amount of genetic material loss. There are few studies in the literature that report on the clinical follow-up and detailed molecular and cytogenetic evaluation of patients with ring chromosomes, which could help establish anticipatory guidelines for these patients [2]. Regarding patients with r(15), there are only two previous reports detailing long-term follow-up [3,4]. We report here on the 20-year clinical and cytogenetic follow-up of a patient with r(15).

## Case presentation

Our patient is the third child of non-consanguineous, healthy parents and has four brothers. Her mother is Caucasian and her father is Asian. At birth her measurements were: weight of 2050g (<fifth centile); length of 48cm (fifth centile); and head circumference (HC) of 30cm (<2standard deviations SD). The first genetic evaluation at eight years and 10 months of age showed her height to be 106cm (<fifth centile); weight 17kg (<fifth centile); and HC 48cm (<2SD). She presented with microcephaly, brachycephaly, high forehead, exotropia, hypoplastic alae nasi, high palate, retrognathism, hyperextensible knees, rough and dry skin of her lower limbs, generalized hirsutism, four café-au-lait spots, disseminated small hypochromic spots and dorsolumbar scoliosis. She had mild intellectual disability, a reduced verbal repertoire, and a docile and cooperative personality.

 At 27 years and 3 months of age, our patient’s mother reports that her daughter had compulsive overeating, an aggressive mood when challenged, and could not read or write. At this time, her measurements were: weight 58kg; height 146.5cm (<third percentile); HC 51.5cm (<2 SD); and a body mass index of 27.23 (+1 SD), corresponding to overweight. In addition to the previous clinical features described above she presented with centripetal obesity, sparse and thin hair, exotropia of her left eye, a thin upper lip, hypoplastic thumbs with proximal implantation, disseminated hypopigmented leaf-shaped spots, acanthosis nigricans, dry skin, subclinical hypothyroidism and regular menstrual cycles since 14 years of age.

**Figure 1:**
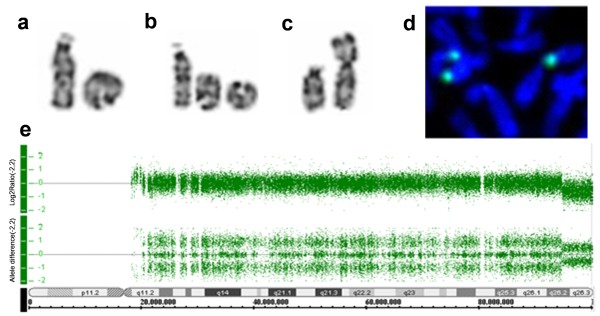
**Partial Giemsa chromosome banding stain (G-banding) karyotype.** Showing one normal chromosome 15 and (**a**) one ring chromosome 15; (**b**) two monocentric ring chromosomes 15; (**c**) one open ring chromosome 15; (**d**) fluorescent *in situ* hybridization result with a centromeric probe showing one dicentric ring chromosome 15; and (**e**) representation from Genotyping Console™ Software 3.0 (Affymetrix Inc., Santa Clara, CA, USA) showing a terminal deletion of chromosome 15. The results are shown for log 2 ratio (a measure of chromosomal copy number; y-axis) versus genomic position on chromosome 15 (HapMap samples).

 A chromosome analysis was first performed in 1989, when our patient was eight years old, from 72-hour lymphocyte cultures according to standard procedures. Giemsa chromosome banding stain (G-banding) revealed a ring chromosome 15 substituting one normal chromosome 15 (Figure 1a). Centromere banding stain (C-banding) and silver staining showed that the ring was monocentric and nucleolar organizer region-positive. Our patient´s parents presented with normal karyotypes. At the age of eight years [5] and 28 years old, an analysis in our patient of 300 metaphases showed 4.7% (in 1989) and 7.7% (in 2009) of cells with loss of the ring or secondary aberrations (two monocentric rings, interlocked rings, dicentric ring, broken ring or open ring) (Figure 1b, c).

 In 2009, we performed fluorescent *in situ* hybridization using a centromeric probe for chromosome 15 (Cytocell Technologies Ltd., Cambridge, UK) and array (Figure 1d-e). Deoxyribonucleic acid (DNA) was isolated from peripheral blood using a Gentra® Puregene® Kit (Qiagen Sciences Inc., Germantown, MD, USA). Samples were genotyped using the Affymetrix® Genome-Wide Human SNP (single-nucleotide polymorphism) Nsp/Sty 6.0 array (Affymetrix Inc., Santa Clara, CA, USA) as previously described [6]. The array data were analyzed using annotation GRCh36/hg18. The resulting karyotype was described as 46,XX,r(15)(p13q26.2)dn.arr 15q26.2q26.3(94,810,000-100,338,900)×1, indicating a 5.5Mb deletion in 15q26.2 (Figure 1e).

## Discussion

We described the case of a patient with a r(15) who was followed-up for 20 years. Three other patients, previously reported by Fryns *et al*. [3], were followed-up for a period of six to nine years, two of them from birth. These patients presented with growth delay without obvious dysmorphism, and their facial characteristics became milder along the years. In the oldest girl described by these authors, puberty developed normally and her menstrual cycle was regular, as found in our patient. The intelligence quotients of their patients remained stable at the different ages evaluated; verbal disabilities and speech difficulties were pronounced around the age of four to six years old, but problems disappeared completely a few years later. Social adaptation and integration were completely normal, and some minor problems such as attention deficit and hyperactivity regressed before the age of seven years old. Another patient with r(15), but with chromosome mosaicism, was followed-up for 29 years by Smith *et al*. [4], who observed that clinical development and ring stability in different tissues, both *in vitro* and *in vivo*, remained stable over the years.

 The patient with r(15) reported here presents some of the characteristics described above, such as microcephaly, bilateral clinodactyly of her fifth finger, café-au-lait and small hypochromic spots spread over her face and the front of her chest and abdomen, and a reduced verbal repertoire. About 12 cases have been reported with pure distal deletion 15q26 [7]. Patients with distal deletion 15q present evident pre- and postnatal growth delay, microcephaly, abnormal ears and face, micrognathia, high palate, renal abnormalities, hypoplastic lungs, slow development, diaphragmatic hernia and complex cardiac malformations. A variety of other characteristics have been reported in association with deletion 15q26, such as café-au-lait spots on the skin, hypertelorism, hypotonia, eye abnormalities, brachydactyly and clinodactyly [7-9]. Some of the features observed in patients with r(15) are the same in patients with 15q terminal deletion. Our patient with r(15) lost 25 genes of 15q according to the University of California Santa Cruz Genome browser (http://genome.ucsc.edu/cgi-bin/hgGateway). The loss of the *IGF1R* gene located at 15q26.3 may be responsible for her growth delay, commonly observed in patients with a deletion in the 15qter and r(15), since it is required for normal embryonic and postnatal growth [10-12]. The *IGF1R* gene also plays an important role in carbohydrate metabolism and central nervous system development [13,14]. It is supposed that the loss of one copy of this gene could also explain the growth delay observed in patients with a deletion of 15q26 [15,16]. Discrete growth delay is a common feature associated with any autosomal ring chromosome and has been attributed to the ring instability, which causes a high frequency of ring loss or duplication, resulting in cell death [17]. In the case of r(15), the growth delay is much more evident due to the loss of an *IGF1R* gene [15-18].

In our study, we observed dynamic mosaicism upon cytogenetic analysis that was not identified by array technique considering its resolution for mosaicism detection [19].

The mosaicism level of the ring chromosome, the variation of tissue-specific mosaicism, the mitotic instability of ring chromosomes and the parental origin of the r(15) have also been proposed as contributing factors for phenotype determination [4,15].

## Conclusion

Both the literature and our data suggest that the wide spectrum of clinical signs and symptoms reported in patients with r(15), extending from a near-normal phenotype to multiple malformations, may result from the interaction of several factors. These include the variation in the amount of euchromatin loss from the short and/or long arm, somatic mosaicism due to ring instability, and epigenetic factors. The latter might relate to the phenotype of the patients because the genomic architecture changes due to the circularization of the chromosome may cause a position effect and an altered expression of genes present in the ring chromosome.

## Consent

Written informed consent was obtained from the patient for publication of this manuscript and accompanying images. A copy of the written consent is available for review by the Editor-in-Chief of this journal.

## Competing interests

The authors declare that they have no competing interests.

## Authors’ contributions

RSG performed cytogenetic and fluorescent *in situ* hybridization studies and wrote the paper. VFAM and DB collected the clinical data of the patient at age 8 and 28 years. RP performed array and analyzed the results. SST performed cytogenetic studies. LDK and MIM coordinated the study and helped to draft the manuscript. All authors read and approved the final manuscript.
